# Propagation phasor approach for holographic image reconstruction

**DOI:** 10.1038/srep22738

**Published:** 2016-03-11

**Authors:** Wei Luo, Yibo Zhang, Zoltán Göröcs, Alborz Feizi, Aydogan Ozcan

**Affiliations:** 1 Electrical Engineering Department, University of California, Los Angeles, CA, 90095, USA; 2 Bioengineering Department, University of California, Los Angeles, CA, 90095, USA; 3 California NanoSystems Institute (CNSI), University of California, Los Angeles, CA, 90095, USA; 4 Department of Surgery, David Geffen School of Medicine, University of California, Los Angeles, CA, 90095, USA

## Abstract

To achieve high-resolution and wide field-of-view, digital holographic imaging techniques need to tackle two major challenges: phase recovery and spatial undersampling. Previously, these challenges were separately addressed using phase retrieval and pixel super-resolution algorithms, which utilize the diversity of different imaging parameters. Although existing holographic imaging methods can achieve large space-bandwidth-products by performing pixel super-resolution and phase retrieval sequentially, they require large amounts of data, which might be a limitation in high-speed or cost-effective imaging applications. Here we report a propagation phasor approach, which for the first time combines phase retrieval and pixel super-resolution into a unified mathematical framework and enables the synthesis of new holographic image reconstruction methods with significantly improved data efficiency. In this approach, twin image and spatial aliasing signals, along with other digital artifacts, are interpreted as noise terms that are modulated by phasors that analytically depend on the lateral displacement between hologram and sensor planes, sample-to-sensor distance, wavelength, and the illumination angle. Compared to previous holographic reconstruction techniques, this new framework results in five- to seven-fold reduced number of raw measurements, while still achieving a competitive resolution and space-bandwidth-product. We also demonstrated the success of this approach by imaging biological specimens including Papanicolaou and blood smears.

High-resolution wide-field optical imaging is needed in various fields, especially in medical and engineering applications that demand large space-bandwidth-products. Originally invented for electron microscopy^1^, holography has become an emerging solution for high-resolution and wide-field digital imaging. The concept of holography relies on reconstructing the image of a specimen using interference patterns created by the diffracted object fields, which can be recorded and digitized even without the use of any lenses. Recent advances in digital holographic microscopy have largely benefited from the rapid evolution of e.g., the opto-electronic sensor technology and computing power^2^, which have led to the development of various new imaging configurations and reconstruction techniques^3^,^4^,^5^,^6^,^7^,^8^,^9^,^10^,^11^,^12^,^13^,^14^,^15^,^16^,^17^,^18^,^19^,^20^,^21^,^22^,^23^,^24^,^25^,^26^,^27^,^28^,^29^,^30^,^31^,^32^,^33^,^34^,^35^.

Generally speaking, in-line holographic imaging modalities, where the diffracted object field and the reference wave co-propagate along the same direction are more susceptible to twin image noise that arises due to the loss of the optical phase or intensity-only spatial sampling at the sensor chip. Although off-axis holography offers a robust solution for this phase retrieval problem by using an angled reference wave, it sacrifices the space-bandwidth-product of the imaging system. For wide-field implementations of high-resolution holographic microscopy, another limitation is posed by pixelation of the holograms since high magnification optics (e.g., objective lenses) or fringe magnification in the form of large distance wave propagation would both result in a significant reduction in the imaging volume and the field-of-view of the microscope.

Previously, these challenges of spatial aliasing (i.e., undersampling) and twin image noise in digital holography have been addressed by pixel super-resolution and phase retrieval techniques, implemented sequentially to reconstruct images of the specimen with ultra-large space-bandwidth-products^16^,^25^,^36^,^37^,^38^. Conventional pixel-super resolution relies on digital synthesis of high spatial frequency content of the sample using multiple low-resolution measurements that are recorded at different sub-pixel displacements between the image sensor and object planes^16^,^21^,^22^,^39^,^40^,^41^,^42^,^43^. Using this mathematical framework, high-resolution (i.e., super-resolved) holograms can be obtained, and then used for digital phase retrieval. To retrieve the lost optical phase in an in-line imaging geometry, multiple super-resolved holograms can be utilized at e.g., different sample-to-sensor distances^25^,^36^,^37^,^44^, illumination angles^33^, or illumination wavelengths^45^,^46^,^47^,^48^. Each one of these holograms essentially serve as independent physical constraints on the amplitude of the optical field, which enables the use of an iterative algorithm to force the complex object field to be consistent with all these measurements^36^,^37^,^44^,^49^,^50^. Although this sequential implementation of pixel super-resolution followed by phase retrieval has enabled digital holographic microscopy to deliver high-resolution and wide-field reconstructions with giga-pixel level throughput, they currently require large amounts of holographic data. For instance, in a multi-height configuration (i.e., using multiple sample-to-sensor distances)^25^,^36^,^37^,^44^, if 4 × 4 pixel super-resolution is implemented at eight different heights, the total number of raw holograms to be captured becomes 128, which could be a limitation for e.g., high-speed imaging applications.

Here, we present a new computational method, termed as *propagation phasor approach*, which for the first time, combines pixel super-resolution and phase retrieval techniques into a unified mathematical framework, and enables new holographic image reconstruction methods with significantly improved data efficiency, i.e., using much less number of raw measurements to obtain high-resolution and wide-field reconstructions of the specimen. Based on our analytical derivations, the twin image noise and spatial aliasing signals, along with other digital holographic artifacts, can be interpreted as noise terms modulated by digital *phasors*, which are all analytical functions of the imaging parameters including e.g., the lateral displacement between the hologram and the sensor array planes, sample-to-sensor distance, illumination wavelength, and the angle of incidence. Based on this new *propagation phasor approach*, we devised a two-stage holographic image reconstruction algorithm that merges phase retrieval and pixel super-resolution into the same unified framework. Compared to previous holographic reconstruction algorithms, our new method reduces the number of raw measurements by five to seven fold, while at the same time achieving a competitive spatial resolution across a large field-of-view.

Based on the same propagation phasor framework, we also created two new digital methods to achieve pixel super-resolution using (1) the diversity of the sample-to-sensor distance (i.e., multi-height based pixel super-resolution), and (2) the diversity of the illumination angle (i.e., multi-angle based pixel super-resolution). We demonstrated the success of these methods by imaging biological specimens such as Papanicolaou and blood smears. We believe that with its significantly improved data efficiency, this new propagation phasor based approach could be broadly applicable to increase the space-bandwidth-product of various digital holographic microscopy systems.

## Methods

### Optical setup for holographic imaging

**Figure 1:**
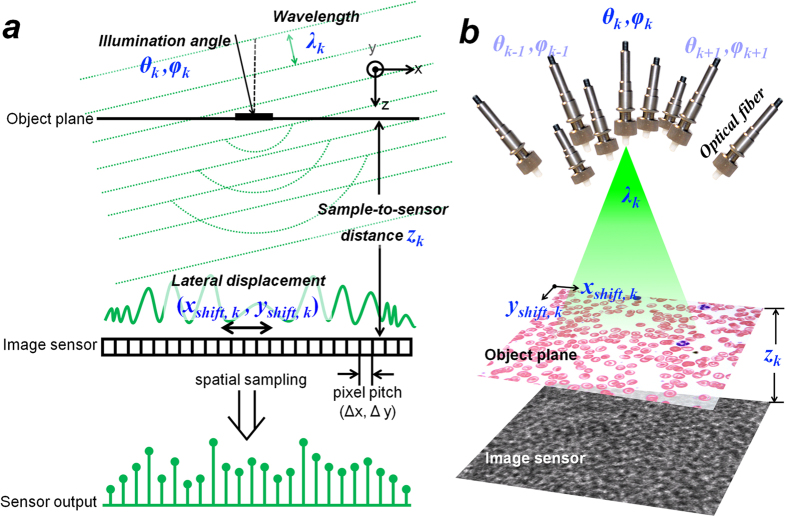
(**a**) Configuration of an in-line holographic imaging system. Some of the controllable parameters of the imaging system are marked in blue color, including the illumination angle (*θ*_*k*_ and *φ*_*k*_), wavelength *λ*_*k*_, sample-to-sensor distance *z*_*k*_, and the lateral displacements between the hologram and the image sensor planes (*x*_*shift,k*_ and *y*_*shift,k*_). (**b**) Schematic of the optical setup of a lensfree on-chip holographic microscope.

Figure 1a depicts our configuration of an in-line holographic imaging system: the coherent or partially coherent incident light first impinges on the specimen, the directly-transmitted light and the scattered light then interfere at an image sensor chip, which samples and digitizes the intensity of this interference pattern. To demonstrate our propagation phasor approach for holographic image reconstruction, we selected to implement it using lensfree holographic microscopy although it is broadly applicable to other holographic microscopy platforms. As depicted in Fig. 1b, our lensfree holographic microscope includes three parts: a fiber-coupled wavelength-tunable light source (WhiteLase-Micro, model VIS, Fianium Ltd, Southampton, UK), an image sensor chip (IU081, Sony Corporation, Japan), and a thin specimen mounted above the sensor chip. The optical fiber’s outlet is placed at e.g. ~10 cm away from the sample whereas the sample-to-sensor distance is typically 0.1–1 mm and thus the illumination at the object plane can be considered as a plane wave. By bringing sample close (sub-mm) to an image sensor chip, lensfree on-chip holography allows the utilization of the image sensor active area as the object field-of-view, creating a unit magnification in-line holographic imaging system, where the spatial resolution and field-of-view can be independently controlled and adjusted by the pixel design and the number of pixels, respectively^38^. The fiber optic cable is mounted on a rotational arm (PRM1Z8, Thorlabs, New Jersey, USA) that can move across a dome above the specimen so that the incidence light can also be adjusted to an arbitrary angle. The rotational arm is loaded on a mechanical linear stage that moves in lateral directions to introduce sub-pixel displacements between the hologram and the image sensor-array. The specimen is held by a piezo-driven positioning stage (MAX606, Thorlabs, New Jersey, USA), which can move vertically to change the distance between the sample and the image sensor chip. During the holographic data acquisition, the tunable source, the mechanical stages, and the image sensor chip are all automated and coordinated by a PC running a custom-written LabVIEW program (Version 2011, National Instruments, Texas, USA).

### Sample preparation

Besides a standard 1951 USAF resolution test target, we also demonstrated the success of our propagation phasor approach by imaging biological samples, including unstained Papanicolaou (Pap) smear slides and blood smears. For this purpose, we used existing and anonymous specimen, where any subject related information cannot be retrieved. Pap smears are prepared using ThinPrep**®** method (Hologic, Massachusetts, USA). The blood smear samples are prepared using EDTA (ethylenediaminetetraacetic acid) anticoagulated human blood and stained with Wright’s Stain^51^.

### Mathematical formalism of propagation phasor approach in digital holography

In this sub-section we present the concept of propagation phasors by deriving the analytical expressions that contain not only the holographic information of the specimen, but also the twin image noise, spatial aliasing signal, and upsampling related spatial artifacts. In this manuscript, we use lower case letters to represent the functions in spatial domain, and the upper case letters for functions in spatial frequency domain. Throughout our analysis, we assume a plane wave illumination as also supported by our imaging set-up, Fig. 1. The transfer function of the optical system between the specimen and the image sensor plane can be written as *h*_*k*_(*x, y, z*_*k*_*, λ*_*k*_*, θ*_*k*_, *φ*_*k*_), where *x* and *y* are the lateral coordinates at the sensor plane, *z*_*k*_ is the vertical sample-to-sensor distance, *λ*_*k*_ is the illumination wavelength, and (*θ*_*k*_, *φ*_*k*_) defines the angle of incidence. The subscript *k* denotes different imaging configurations, achieved by e.g., vertically moving the specimen or sensor chip to record the holograms at different sample-to-sensor distances *z*_*k*_, changing the illumination wavelength *λ*_*k*_, or tilting the illumination beam to change the angle of incidence, *θ*_*k*_ and *φ*_*k*_. One additional pair of variables in our imaging configuration is the lateral displacements between the image sensor and the object planes, i.e., *x*_*shift,k*_ and *y*_*shift,k*_, see Fig. 1a. Such sub-pixel displacements are utilized as one way of mitigating the spatial undersampling at the image sensor chip due to a large pixel size.

Under these different imaging configurations, each labeled with index *k*, the transmission properties of a two-dimensional (2D) specimen can be generally expressed as *o*_*k*_(*x, y*) = 1 + *s*_*k*_(*x, y*), where *s*_*k*_ refers to the scattered object field that interferes with the background unscattered light. The frequency spectrum *O*_*k*_(*f*_*x*_*, f*_*y*_) of *o*_*k*_(*x, y*) can be written as:


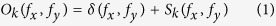


Similarly, we can write the 2D spatial frequency spectrum of the transfer function *h*_*k*_(*x*, *y*, *z*_*k*_, *λ*_*k*_, *θ*_*k*_) as:


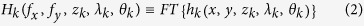


where FT refers to the Fourier Transform operation. From now on, we will simplify the expressions of all the frequency spectra in our equations by hiding the spatial frequency variables *f*_*x*_, and *f*_*y*_. The frequency spectrum of the field intensity *i*_*k*_(*x*, *y*) on the image sensor plane can then be expressed as:


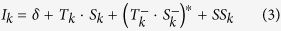


where ‘·’ represents the multiplication operation, the superscript ‘−’ represents using variable set (−*f*_*x*_, −*f*_*y*_) instead of (*f*_*x*_, *f*_*y*_) and the asterisk stands for complex conjugate operation. *SS*_*k*_ represents the self-interference terms, which can be written as *SS*_*k*_ = Γ_*fx,fy*_{*H*_*k*_ · *S*_*k*_}, where Γ_*fx,fy*_ refers to the autocorrelation operation. *T*_*k*_ is determined by the transfer function *H*_*k*_, i.e.,:


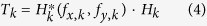


where *f*_*x,k*_ = *n_k_* · sin *θ*_*k*_·cos φ_*k*_/*λ*_*k*_, *f*_*y,k*_ = *n*_*k*_ · sin *θ*_*k*_ · sin φ_*k*_/*λ*_*k*_, and *n*_*k*_ is the refractive index of the medium, which is assumed to be a function of only the illumination wavelength. It is important to notice that *H*_*k*_ is a complex function with a unit magnitude, defining a *phasor*^52^. Based on Eq. (4), as a product of 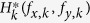 and *H*_*k*_, the function *T*_*k*_ is also a *phasor*, and we term *T*_*k*_ as a ***propagation phasor***, the function of which in our reconstruction framework will be more clear later on.

When any intensity distribution *i*_*k*_(*x*, *y*) is sampled by an image sensor-array with a pixel pitch of Δ*x* and Δ*y* in lateral directions, the discrete Fourier transform (DFT) of the sensor’s output can be expressed as:


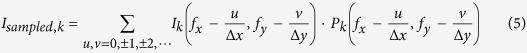


In Eq. (5) *u* and *v* are integers representing the aliasing orders, and (*u*, *v*) = (0, 0) denotes the non-aliased *target* signal of the object. *P*_*k*_(*f*_*x*_, *f*_*y*_) is the 2D FT of the pixel function that defines the responsivity distribution within each pixel of the image sensor chip^30^. Originally, *f*_*x*_, and *f*_*y*_ in Eq. (5) are discrete frequency values confined within the Nyquist window. Based on the periodic nature of DFT, Eq. (5) and all of our further derivations can be numerically extended to a broader frequency domain by simply upsampling the raw measurements. Therefore, without change of notations, *I*_*sampled,k*_ refers to the DFT of the *upsampled* version of our raw measurements.

Now we will incorporate the lateral displacements between the holograms and the image sensor chip into Eq. (5). If we add lateral shifts (*x*_*shift,k*_, *y*_*shift,k*_) to each hologram, then Eq. (5) can be re-written as:


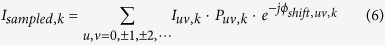


where we simplify the expression of spatial aliasing order by using the subscript *uv*, and *ϕ*_*shift,uv,k*_ represents the phase change caused by a lateral shift:


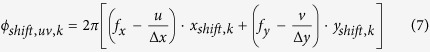


In Eq. (6), by replacing the expression of *I*_*uv,k*_ with Eq. (3), we can obtain an expanded expression for *I*_*sampled,k*_:


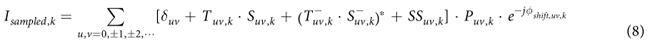


On the right side of Eq. (8), we can see that, for each aliasing order (i.e., each combination of *u* and *v*, including the target signal: *u* = 0, *v* = 0), there are four items inside the square brackets. *The first item, δ*_*uv*_*, represents the background light, the second item, T*_*uv,k*_* · S*_*uv,k*_*, represents the real image, the third item*, 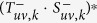
*, represents the twin image; and the last item, SS*_*uv,k*_*, is the self-interference term*.

In the next sub-sections, we will present a generic, two-stage holographic reconstruction algorithm using propagation phasors, which aims to recover the object term *δ*_00_ + *S*_00,*k*_ from a series of measured holograms.

### Stage I of Propagation Phasor based Holographic Reconstruction: Generation of an Initial Guess

**Figure 2:**
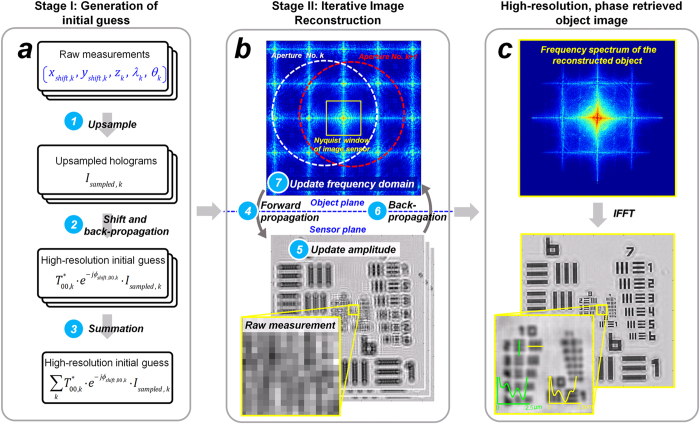
Propagation phasor approach-based holographic image reconstruction. (**a**) Stage I: generation of an initial guess. (**b**) Stage II: iterative image reconstruction. (**c**) High-resolution, phase retrieved reconstruction of the object.

As depicted in Fig. 2, the first stage of the reconstruction is to generate a high-resolution initial guess of the specimen, and this *Stage I* is composed of three steps (i.e., Steps 1–3 in Fig. 2).

Step 1: Upsampling of each raw measurement serves as the first step in our holographic reconstruction algorithm. This upsampling factor, although does not introduce any new information, should be large enough to expand the expression of *I*_*sampled,k*_ to cover the entire passband of the optical system. Since the computation cost of the reconstruction increases quadratically with the upsampling factor, it should also be limited to avoid unnecessary computational burden/time. For our lensfree microscopy platform reported here, we typically set an upsampling factor of ≤7.

Step 2: The second step of the holographic reconstruction is to offset the lateral displacements *x*_*shift,k*_, and *y*_*shift,k*_, and then perform back-propagation on the upsampled raw measurements. To do so, we multiply both sides of Eq. (8) with 
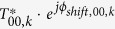
 and reorganize the terms to extract the true object signal, i.e., the target signal:


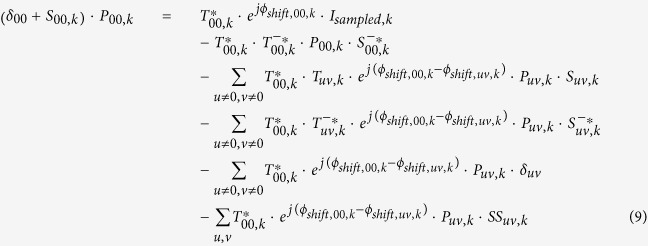


On the left side of Eq. (9), we have kept the pixel function *P*_00,*k*_ multiplied with *δ*_00_ + *S*_00,*k*_; note, however, that it can be later removed using deconvolution techniques as the last step of the holographic reconstruction^30^. The right side of Eq. (9) shows that in order to extract(*δ*_00_ + *S*_00,*k*_ ·* P*_00,*k*_, there are five terms that need to be eliminated from the back-propagated intensity (i.e., 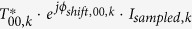). The first term, 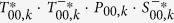, represents the *twin image noise*; the second and third terms which contain *S*_*uv,k*_ or 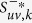
(*u* ≠ 0, *v* ≠ 0) represent the *spatial aliasing signals* for real and twin images, respectively; the fourth term with *δ*_*uv*_ (*u* ≠ 0, *v* ≠ 0) is the *high frequency artifacts* generated during the upsampling process. The last term with *SS*_*uv,k*_ is the *self-interference signal*.

**Figure 3:**
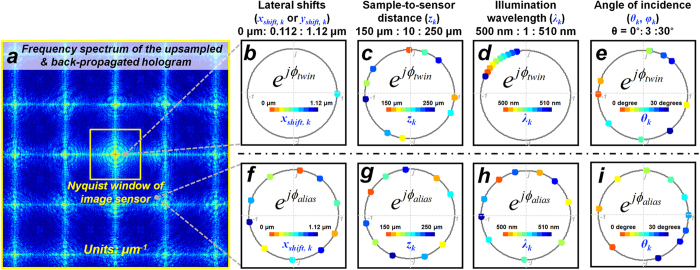
Examples of propagation phasor values as a function of various imaging parameters. (**a**) The frequency spectrum of an upsampled and back-propagated hologram. To give examples, we probe the values of the ***twin image phasor*** at (*f*_*x*_ = 0.1 μm^−1^, *f*_*y*_ = 0 μm^−1^) as shown in (**b–e**) and the ***spatial aliasing phasor*** at (*f*_*x*_ = 0.8 μm^−1^, *f*_*y*_ = −0.8 μm^−1^) as shown in (**f–i**). In each subfigure, we scan one of the imaging parameters while keeping all the others constant, and plot the values of the phasors in color-coded points on the unit circle. The first row shows the twin image phasor values as a function of (**b**) the lateral shifts between the hologram and the image sensor-array, *x*_*shift,k*_ and *y*_*shift*,*k*_, (**c**) the sample-to-sensor-distance, *z*_*k*_, (**d**) the illumination wavelength, *λ*_*k*_, and (**e**) the illumination angle *θ*_*k*_. Similarly, the second row shows the spatial aliasing phasor values as a function of (**f**) *x*_*shift,k*_ and *y*_*shift,k*_, (**g**) *z*_*k*_, (**h**) *λ*_*k*_, and (**i**) *θ*_*k*_.

**Figure 4:**
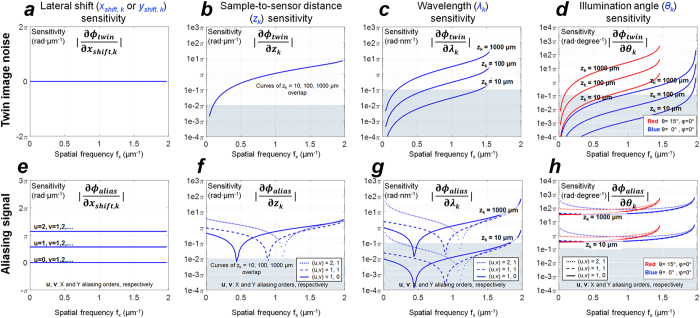
Sensitivity of propagation phasors to various imaging parameters. The first row: the partial derivatives of the twin image phasor’s angle (Φ_*twin*_) with respect to (**a**) the lateral shifts *x*_*shift,k*_ and *y*_*shift,k*_, (**b**) the sample-to-sensor distance *z*_*k*_, (**c**) the illumination wavelength *λ*_*k*_, and (**d**) the illumination angle *θ*_*k*_. The second row: the partial derivatives of the twin image phasor’s angle (Φ_*alias*_) with respect to (**e**) *x*_*shift,k*_ and *y*_*shift,k*_, (**f**) *z*_*k*_, (**g**) *λ*_*k*_, and (**h**) *θ*_*k*_.

Step 3: Summation of all the upsampled and back-propagated holograms 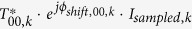 to generate an initial guess. This initial summation can greatly suppress the twin image noise, aliasing signal and other artifact terms outlined above in Step 2. To better explain the impact of this summation step, we can simplify the expression of the phasor terms in Eq. (9) as:


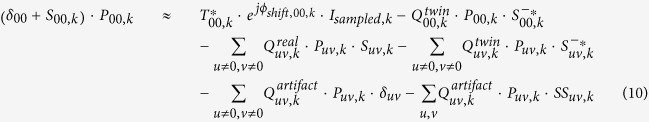


where 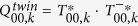, 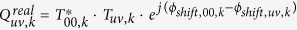, 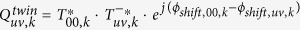, and 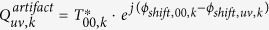. Here, 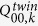, 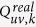, 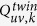 and 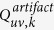 are also *phasors* with unit amplitudes, and their phases change as a function of all the imaging parameters (i.e., *z*_*k*_, *λ*_*k*_, *θ*_*k*_, *φ*_*k*_, *x*_*shift,k*_, and *y*_*shift,k*_), see e.g., Figs 3 and 4.

Also notice that except the illumination wavelength *λ*_*k*_, the changes of the imaging parameters *z*_*k*_, *θ*_*k*_, *φ*_*k*_, *x*_*shift,k*_, and *y*_*shift,k*_ do not affect the transmission properties of the 2D specimen. During the imaging process, we confine the illumination wavelengths within a narrow spectral range, typically less than 10 nm, so that the transmission properties of the specimen and the image sensor’s pixel function can be approximately considered identical when generating an initial guess of the object, i.e., *S*_*uv,k*_ ≈ *S*_*uv*_, and *P*_*uv,k*_ ≈ *P*_*uv*_. If we list Eq. (10) for all the possible *K* imaging conditions (e.g., as a function of various illumination wavelengths, sub-pixel shifts, etc.), and then sum them up with a set of weighting factors, {*c*_*k*_}, we can have:


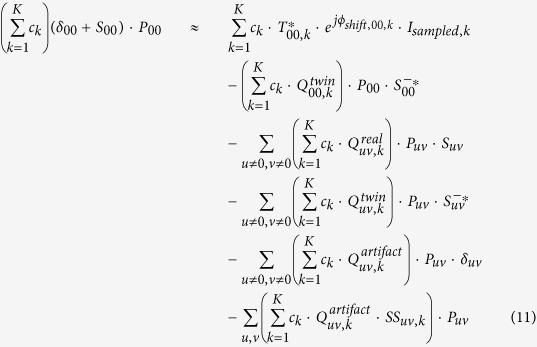


By finding a set of weighting factors {*c*_*k*_} that satisfy 
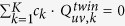
(*u,v* = 0, ±1, ±2,···); 
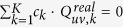
 (*u* ≠ 0, *v* ≠ 0); 
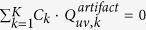
 (*u* ≠ 0, *v* ≠ 0); and 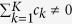, we can have “*complete elimination”* of the twin image noise, aliasing signals and upsampling related spatial artifacts, while still maintaining the target object function, (*δ*_00_ + *S*_00_)·*P*_00_. However, considering the fact that 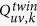, 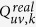 and 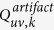 are also functions of spatial frequencies (*f*_*x*_*, f*_*y*_), it is computationally expensive to obtain a set of ideal {*c*_*k*_} values. Therefore we adopt an alternative strategy as shown in Fig. 2 to create our initial object guess and set all {*c*_*k*_} values to 1, and directly sum up the upsampled and back-propagated holograms, 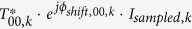. After this summation, the left side of Eq. (11) becomes K·(*δ*_00_ + *S*_00_ · *P*_00_), while on the right side, the summations of the phasors 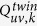, 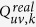 and 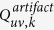 follow:


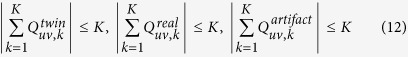


In fact, as illustrated in Fig. 3, with proper selection of the imaging configuration, the summations of these phasors can be significantly smaller than *K*. This implies that, by simply summing up Eq. (11) for all *K* imaging configurations, the twin image noise 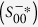, aliasing signals (*S*_*uv,k*_ and 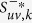, *u* ≠ 0,*v* ≠ 0) and upsampling related artifacts 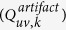 can be significantly suppressed in comparison with the target signal (*δ*_00_ + *S*_00_) · *P*_00_. Therefore, we consider a simple summation as a good *initial guess* of the specimen at this Stage I of our propagation phasor based holographic reconstruction approach, i.e.,


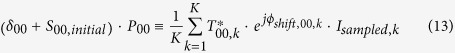


This initial guess is then used as the input to an iterative algorithm (*Stage II*) to reconstruct and refine the object function/image, which will be detailed in the next sub-section.

### Stage II of Propagation Phasor based Holographic Reconstruction: Iterative Image Reconstruction

Using the initial guess defined by Eq. (13), we next implement an iterative process as the second stage of our propagation phasor based holographic reconstruction algorithm to eliminate the remaining twin image noise, aliasing signal, and the upsampling related artifacts. Each iteration of *Stage II* is comprised of four steps (i.e., Steps 4 through 7-see Fig. 2):

Step 4: Based on the parameters of each illumination condition, (i.e., *z*_*k*_*, λ*_*k*_*, θ*_*k*_*, φ*_*k*_), we apply a phase modulation on the initial guess of the specimen, defined by Eq. (13), and propagate the field from the object plane to the image sensor using the angular spectrum approach^52^. For this wave propagation, we use the free space transfer function:


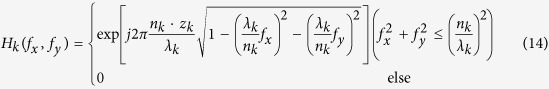


We term the wave propagation from the object plane to the image sensor as forward-propagation, and denote the spatial form of the forward-propagated field as *g*_*forward,k*_(*x, y*). Note that the Fresnel transform based digital wave propagation can also be used at this step, although for high-resolution imaging applications the angular spectrum approach is more suitable without any low NA approximations.

Step 5: On the image sensor plane, we use the raw measurements (i.e., the low-resolution, undersampled holograms) to update the amplitude of the high-resolution, forward-propagated field *g*_*forward,k*_(*x, y*). To do so, we first convolve the intensity of the field, | *g*_*forward,k*_(*x, y*)|^2^, with the pixel function of the image sensor^30^, and shift the convolved intensity by an amount of (*x*_*shift,k*_, *y*_*shift,k*_) to compensate the corresponding lateral displacement. Next, this shifted intensity is downsampled to the same resolution as the raw measurement, and the difference between this downsampled intensity and the raw measurement is considered as a low-resolution correction map. In order to apply this low-resolution correction map to each shifted intensity, we upsample this correction map by taking its Kronecker product with the pixel function, and add the upsampled correction map to the shifted intensity with a relaxation factor (typically ~0.5). Then this ‘corrected’ intensity is deconvolved with the pixel function using Wiener deconvolution, and shifted back in place by the amount of (−*x*_*shift,k*_, −*y*_*shift,k*_). The Wiener filter takes into account the measured noise level of the image sensor to avoid over-amplification of noise during each iteration. We then use the square root of the deconvolved and shifted intensity to replace the amplitude of *g*_*forward,k*_(*x, y*), while keeping its phase *unaltered*.

Step 6: Back-propagate the amplitude-updated, high-resolution field to the object plane, and remove the phase modulation caused by the illumination angle.

Step 7: The back-propagated field is then used to update the transmitted field on the object plane. Different from Step 6, this update on the object plane is carried out in the spatial frequency domain. The spatial frequency region for this update is a circular area centered at *f*_*x,k*_ = *n_k_* · sin *θ*_*k*_·cos φ_*k*_/*λ*_*k*_, *f*_*y,k*_ = *n_k_* · sin *θ*_*k*_·sin φ_*k*_/*λ*_*k*_, and we choose the radius of the circle so that all the spatial frequencies within it experience less than 3dB amplitude attenuation during wave propagation. This update in the spatial frequency domain is also smoothened using a relaxation factor of ~0.5. In other words, the updated frequency region is the weighted sum of the old transmitted field and the back-propagated field, and the weighting factor (i.e., relaxation factor) for the back-propagated field is ~0.5. After this update, we convert the phase of the field into an optical path length map of the object, and the amplitude of the field gives us the object’s final transmission image, i.e., reconstruction. Note that for relatively thick specimen, phase unwrapping needs to be performed before converting the reconstructed phase into an optical path length^35^.

These above outlined steps (Steps 4 to 7) are performed for every imaging configuration. It is considered as one iteration cycle when all the *K* raw measurements are used for once. Similar to the convergence condition defined in Ref. 53, we determine the convergence of our iterations and the reconstruction when the sum-squared error (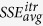) between the raw measurement and the downsampled intensity map satisfies the following criterion:


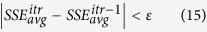


where ‘*itr’* is the index of the iteration cycle, and *ε* is the convergence constant, empirically defined as ~0.2% of 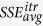.

### Computation platform used for propagation phasor based holographic reconstructions

**Figure 5:**
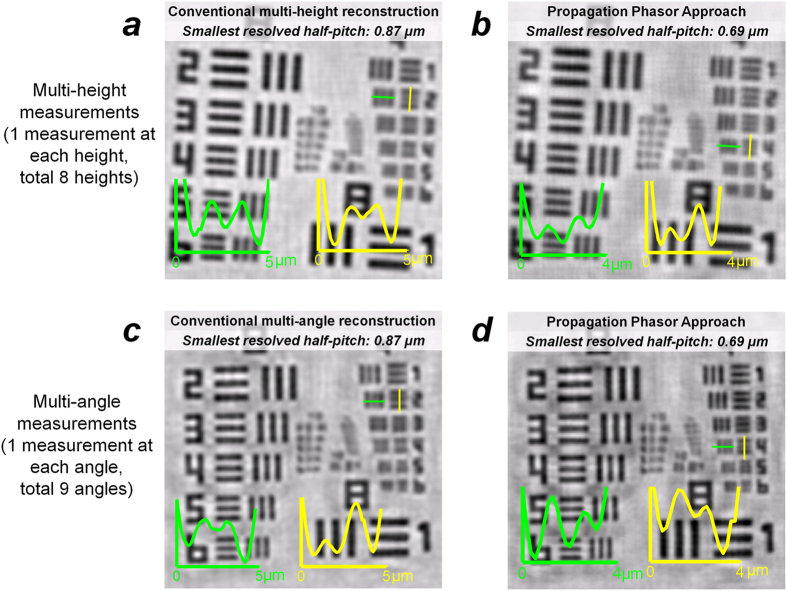
Propagation phasor approach can achieve pixel super-resolution using the diversity of the sample-to-sensor distance and the illumination angle. (**a**) Conventional multi-height based image reconstruction result^25^. At each of the 8 heights (i.e., sample-to-sensor distances, 200 μm: 15 μm : 305 μm), only one raw hologram is used. (**b**) Propagation phasor approach-based reconstruction with improved resolution using the same data set used in (**a**). (**c**) Conventional multi-angle based image reconstruction result^33^. At each of the nine illumination angles (−30° : 15° : 30°, two axes), only one raw hologram is used. (**d**) Propagation phasor approach-based reconstruction with improved resolution using the same data set used in (**c**).

**Figure 6:**
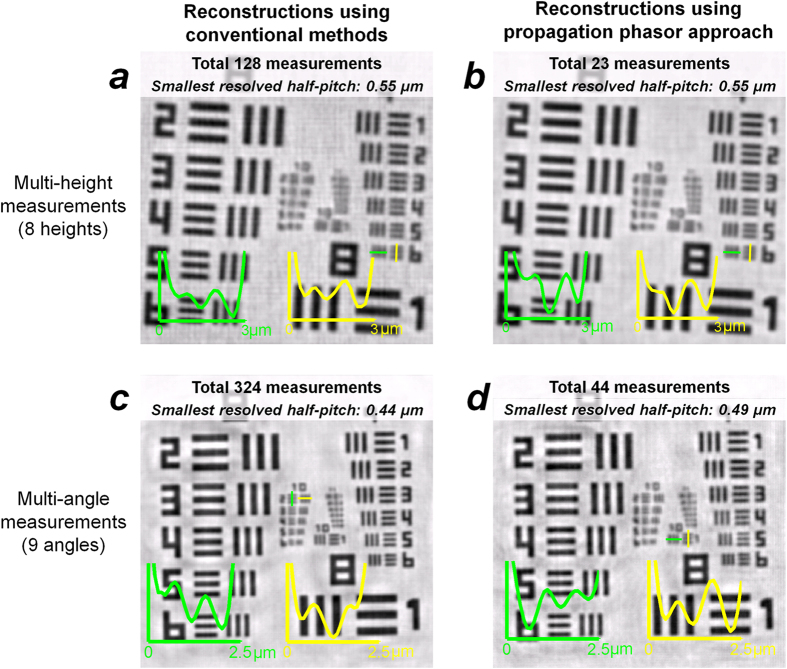
Propagation phasor approach improves the data efficiency of holographic imaging. (**a**) Conventional multi-height based reconstruction result^25^. At each of the 8 heights (200 μm: 15 μm: 305 μm), 16 raw holograms are acquired to generate pixel super-resolved holograms, which results in a total of 128 raw measurements. (**b**) Propagation phasor approach-based reconstruction, using only 23 raw measurements. At only one of the heights (200 μm), 16 raw measurements are used for pixel super-resolution, while at each of the other heights, only one raw measurement is used. (**c**) Conventional synthetic aperture based reconstruction result^33^. At each one of the 9 angles (−30°: 15°: 30°, two axes), 36 raw holograms are acquired to generate pixel super-resolved holograms, which results in a total of 324 raw measurements. (**d**) Propagation phasor approach-based reconstruction, using only 44 raw measurements. At the normal illumination, 36 raw measurements are used for pixel super-resolution, while at each one of the other angles, only one raw measurement is used.

For proof-of-concept implementation, our propagation phasor approach based reconstruction algorithm has been implemented using MATLAB (Version R2012a, MathWorks, Massachusetts, USA) on a desktop computer with 3.60-GHz central processing unit (Intel Xeon E5-1620) and 16 GB random-access memory. Using an upsampling factor of seven, the computation time of one iteration in reconstruction *Stage II* (detailed in the previous sub-section) is ~1.2 seconds for a region-of-interest of ~1 × 1 mm^2^. As for the total computation time including *Stages I* and *II*, assuming that the number of intensity distribution updates is ~8–10 per iteration (see e.g. Fig. 5b,d and Fig. 6b,d), and that the convergence can be reached within ~6–7 iteration cycles, the total image reconstruction time ranges between ~1–1.5 minutes per 1 mm^2^. More than 85% of this computation time is spent on wave propagation between the sample and the image sensor planes, which heavily relies on Fast Fourier Transforms (FFTs). Therefore, the adoption of graphic processing units (GPUs) or other parallel computing architectures could significantly reduce the total computation time^21^.

## Results and Discussion

The main challenges of wide field-of-view, high-resolution holographic imaging include: (1) phase retrieval, and (2) mitigating the undersampling caused by an image sensor chip. *The propagation phasor approach of this manuscript relies on the fact that in the digital hologram of a specimen, the twin image noise and spatial aliasing signals vary under different imaging configurations*. Such variations enable us to eliminate these unwanted noise terms (twin image noise and aliasing signal) and obtain phase-retrieved and high-resolution (i.e., super-resolved) reconstructions of the object. The imaging configuration in a holographic microscope can in general be changed by varying different parameters: (1) the lateral displacements between the holograms and the sensor-array (i.e., lateral relative shifts *x*_*shift,k*_and *y*_*shift,k*_), (2) the sample-to-sensor distance (*z*_*k*_), (3) the illumination wavelength (*λ*_*k*_), and (4) the angle of incidence (*θ*_*k*_*, φ*_*k*_). In this section, to better illustrate the inner workings of our propagation phasor approach, we will first demonstrate the dependencies of the twin image noise and the aliasing signal on these controllable imaging parameters and then explore and summarize the combinations of these imaging parameters that can create phase-retrieved and high-resolution reconstructions while also improving the data efficiency of holographic imaging.

### Dependency of Twin Image Noise and Aliasing Signal on Imaging Parameters

From Eq. (10), we can see that all the terms which need to be eliminated from an upsampled and back-propagated hologram 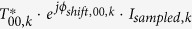 are modulated by phasors, including: (1) the twin image term, modulated by 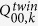; (2) aliasing signals, modulated by 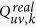 and 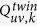, *u* ≠ 0, *v* ≠ 0); (3) upsampling artifacts (*δ*_*uv*_ terms modulated by 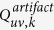
*, u* ≠ 0, *v* ≠ 0); and (4) self-interference patterns (*SS*_*uv,k*_ terms modulated by 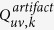). From the perspective of our propagation phasor approach, we desire that the phasors that modulate these unwanted noise terms or artifacts exhibit sufficient variations across [0, 2π], so that they can be significantly suppressed during the initial summation in the reconstruction Stage I (detailed in the Methods Section). In this manuscript, we focus our discussion on twin image phasor 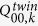 and aliasing related phasors 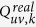, 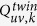, (*u* ≠ 0, *v* ≠ 0), where the conclusions would be broadly applicable to a wide range of holographic imaging systems (lens-based or lensfree). Meanwhile, the self-interference patterns/artifacts are much weaker in signal strength compared to the holographic interference terms and can be easily suppressed by the iterative reconstruction algorithm (Stage II) that is detailed in the Methods Section.

To illustrate the dependencies of the twin image noise and the aliasing signal on the holographic imaging parameters, we choose the twin image phasor 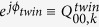 and one of the spatial aliasing phasors, i.e., 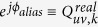 (*u* = 1, *v* = 1), as examples and visualize them as a function of the imaging parameters (*x*_*shift,k*_, *y*_*shift,k*_, *z*_*k*_, *λ*_*k*_, *θ*_*k*_, and *φ*_*k*_) as shown in Fig. 3. In each sub-figure of Fig. 3, we only change one of the imaging parameters while keeping all the others constant. For instance, in Fig. 3b that shows *e*^*j*^^*ϕ_twin_*^, we only change the lateral shift *x*_*shift,k*_ from 0 μm to 1.12 μm (i.e., the pixel pitch of the image sensor chip used in our experiments) with a step size of ~0.11 μm, while the other parameters are fixed at *z*_*k*_ = 150 μm, *λ*_*k*_ = 500 nm, *θ*_*k*_ = 0°, and *φ*_*k*_ = 0°. Similarly, Fig. 3c through Fig. 3e depict *e*^*j*^^*ϕ_twin_*^ as a function of *z*_*k*_, *λ*_*k*_, and *θ*_*k*_ separately, while Fig. 3g through Fig. 3i show *e*^*jϕ_alias_*^ as a function of *x*_*shift,k*_, *z*_k_, *λ*_*k*_, and *θ*_*k*_, respectively.

From Figs 3b–i we can see that, except the twin image phasor’s *insensitivity* to lateral shifts, the diversity of all the other imaging parameters can cause both the twin image phasor and the aliasing phasors to be modulated. To better illustrate these phasors’ sensitivities to various imaging parameters, we calculated in Fig. 4 the partial derivatives of *ϕ*_*twin*_ and *ϕ*_*alias*_ with respect to *x*_*shift,k*_, *y*_*shift,k*_*, z*_*k*_, *λ*_*k*_, *θ*_*k*_ and *φ*_*k*_. Next we will analyze the values of these partial derivatives along the *f*_*x*_ axis (i.e., *f*_*y*_ = 0), and summarize each imaging parameter’s effect on *ϕ*_*twin*_ and *ϕ*_*alias*_ (see Fig. 4a–h).

#### *Lateral shifts* (*x*_*shift,k*_, *y*_*shift,k*_)

Since the twin image phasor 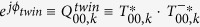 (see Eq. 4) does not contain variables *x*_*shift,k*_ or *y*_*shift,k*_, the absolute value of its partial derivatives with respect to *x*_*shift,k*_ and *y*_*shift,k*_ is zero, i.e., |*∂ϕ*_*twin*_/∂*x*_*shift,k*_| = 0 and |*∂ϕ*_*twin*_/∂*y*_*shift,k*_| = 0 (Fig. 4a). In other words, lateral shifts do *not* introduce any variations in the twin image noise term as a result of which they are not directly useful for twin image elimination or phase retrieval. On the other hand, as illustrated in Fig. 4e, when spatial aliasing exists in either *x* or *y* direction (i.e., *u* ≠ 0, *v* ≠ 0), we then have |*∂ϕ*_*alias*_/∂*x*_*shift,k*_| > 0 and |*∂ϕ*_*alias*_/∂*y*_*shift,k*_| > 0, which suggests that *x*_*shift,k*_ and *y*_*shift,k*_ introduce linear phase modulations (see Eq. 7) in the spatial aliasing phasor term. This linear relationship between *ϕ*_*alias*_ and (*x*_*shift,k*_, *y*_*shift,k*_) makes the lateral shifts ideal choice for aliasing signal elimination. As shown in the Supplementary Materials, if we set the lateral shifts to be *evenly* distributed within one pixel pitch, where *x*_*shift,k*_ ∈{*m*/(*M* · Δ*x*)|*m* = 1,2,…M} and *y*_*shift,k*_ ∈{*n/(N* · Δ*y*)|*n* = 1,2,…N} summing up the upsampled and back-propagated holograms (i.e., Stage I of the reconstruction algorithm detailed in the Methods Section) can lead to *complete elimination of the aliasing signals*. This summation is mathematically equivalent to back-propagating the *pixel super-resolved* holograms^16^,^22^,^25^,^26^,^30^,^31^,^32^,^33^,^40^,^41^,^42^,^43^,^54^,. To conclude, the diversity of the lateral shifts can only contribute to the aliasing signal elimination, i.e., pixel super-resolution.

#### *Sample-to-sensor distance* (*z*_*k*_)

Using the diversity of the sample-to-sensor distance (*z*_*k*_) to eliminate the twin image noise has been one of the most widely-used phase retrieval techniques in holographic image reconstruction^13^,^25^,^27^,^32^,^36^,^37^,^49^,^50^. For completeness of our discussion, here we analyze the effect of *z*_*k*_ on the twin image noise from the perspective of the propagation phasor approach. As shown in Fig. 4b, |*∂ϕ*_*twin*_/*∂z*_*k*_|rises as spatial frequency *f*_*x*_ increases. Except at very low spatial frequencies (e.g., |*f*_*x*_| < 0.1 μm^−1^), *ϕ*_*twin*_ exhibits strong sensitivity to *z*_*k*_. For example, even at |*f*_*x*_| ≈ 0.1 μm^−1^, changing the sample-to-sensor distance by ~100 μm can make the twin image phasor *e*^*j*^^*ϕ_twin_*^ *reverse* its polarity. This sensitivity makes *z*_*k*_ a *very useful variable* for twin image noise elimination. For aliasing signal elimination, as depicted in Fig. 4f, we can see that *ϕ*_*alias*_ also shows a good sensitivity to *z*_*k*_, i.e. |*∂ϕ*_*alias*_/*∂z*_*k*_| ≥ 0.01π except for a very limited number of spatial frequency points. ***Therefore, besides twin image elimination, the diversity of z***_***k***_***can also be used for aliasing signal elimination***.

#### *Wavelength* (*λ*_*k*_)

The diversity of illumination wavelength can be used for twin image elimination (i.e., phase retrieval)^46^,^55^. We have previously reported that it can also be used for eliminating the spatial aliasing signals^35^. As shown in Fig. 4c,g, one important property of |*∂ϕ*_*twin*_/*∂λ*_*k*_| and |*∂ϕ*_*alias*_/*∂λ*_*k*_| is that they show strong dependencies on the illumination wavelength *only when* the sample-to-sensor distance *z*_*k*_ is large enough (e.g., *z*_*k*_ > ~100 μm). Stated differently, by changing the illumination wavelength *λ*_*k*_, the holographic interference patterns at the sensor-array will surely vary, but such variations become more pronounced and useful at larger distances, *z*_*k*_. ***Therefore, in a point-to-point focused imaging system (using e.g., a lens-based imaging set-up), the diversity of wavelength is of no use for phase retrieval or resolution enhancement unless a slight defocus (i.e., z***_***k***_***) is introduced in the imaging system***.

#### *Angle of incidence* (*θ*_*k*_ *, φ*_*k*_)

We have previously reported the use of the diversity of illumination angles (*θ*_*k*_ and *φ*_*k*_) for phase retrieval^16^,^22^,^33^,^38^ as well as for expanding/improving the frequency bandwidth, i.e., the spatial resolution through a synthetic aperture approach in lensfree on-chip microscopy^33^. As shown in Fig. 4d,h, similar to the case of wavelength diversity, to make use of the illumination angle for phase retrieval and elimination of aliasing signal, sufficient sample-to-sensor distance (e.g., *z*_*k*_ > 100 μm) is needed. Fig. 4d also suggests that, for phase retrieval, relatively large angular variations (e.g., Δ*θ* > 10°) are preferred since |*∂ϕ*_*alias*_/*∂θ*_*k*_| > 0.1*π*·degree^−1^. Another important observation from Fig. 4h is that at different illumination angles *θ*_*k*_, |*∂ϕ*_*alias*_/*∂θ*_*k*_| remains non-zero in most of the spatial frequencies, which is similar in behavior to |*∂ϕ*_*alias*_/*∂x*_*shift,k*_|as shown in Fig. 4e. Intuitively, this implies that slight perturbations on the illumination angle will introduce lateral shifts of the interference patterns on the image sensor plane, which can be considered as one method of generating *x*_*shift,k*_, and *y*_*shift,k*_. In fact, shifting the light source by small amounts has been proven as an effective way of performing lateral shift-based pixel super-resolution in lensfree holography^16^,^21^,^22^.

Regarding the parameter *φ*_*k*_, although not depicted in Fig. 4, it is important to emphasize that |*∂ϕ*_*twin*_/*∂φ*_*k*_| = 0 and |*∂ϕ*_*alias*_/*∂φ*_*k*_| = 0 when *θ*_*k*_ = 0, and that the sensitivity of both *ϕ*_*twin*_ and *ϕ*_*alias*_ to *φ*_*k*_ increases with *θ*_*k*_. Therefore, both *θ*_*k*_ and *φ*_*k*_ can be used for the elimination of twin image noise and spatial aliasing signal.

The above-described contributions of various imaging parameters to eliminate twin image noise and spatial aliasing signal terms are summarized in Table 1. From Table 1 we can see that the propagation phasor approach of this manuscript: (***1***) *provides a unique mathematical formalism that combines/merges various existing phase retrieval and pixel super-resolution techniques used in digital holography into the same unified framework*, and (***2***) *creates two new techniques to eliminate the aliasing signal in digital holography, namely using the diversity of the sample-to-sensor distance, and the diversity of the illumination angle*. For consistency with the previous used terminology, we name these two new methods as *multi-height based pixel super-resolution* and *multi-angle based pixel super-resolution*, respectively. Next, we will experimentally demonstrate the imaging results and the advantages of these two new methods.

### Propagation Phasor Approach Using Multi-height and Multi-angle Holographic Data

Using this new propagation phasor based reconstruction framework, the diversities of sample-to-sensor distance or illumination angle can enable not only twin image elimination, but also resolution enhancement, i.e., super-resolution. To demonstrate the resolution enhancement brought by the diversity of *z*_*k*_ (i.e., multi-height based pixel super-resolution – Table 1), we captured the holograms of a standard resolution test target at eight different heights, where the values of *z*_*k*_ are evenly distributed between 200 μm and 305 μm with a spacing of ~ 15 μm. For comparison, we first reconstructed the specimen using a previous technique: multi-height based phase retrieval algorithm^25^,^27^,^32^ (see Fig. 5a). For the same set of raw data, compared to this previous technique our propagation phasor based reconstruction delivers a half-pitch resolution improvement from ~0.87 μm to 0.69 μm, corresponding to a numerical aperture (NA) improvement from 0.3 to 0.4 (wavelength: 530 nm), see Fig. 5b.

In addition to multi-height based pixel super-resolution, a similar resolution enhancement can also be achieved using the diversity of illumination angles (i.e., multi-angle based pixel super-resolution – Table 1). As shown in Fig. 5c,d, we demonstrated multi-angle pixel super-resolution using the data captured from 9 different illumination angles, where one of them is vertical (0°), and rest of the angles are placed at ± 15° and ± 30° along two axes above the specimen (see Fig. 1b). The half-pitch resolution improvement brought by the diversity of illumination angle is also similar: from ~0.87 μm down to 0.69 μm.

In the next sub-section we will demonstrate that much higher resolution images can be reconstructed using our propagation phasor approach by simply adding lateral shift based pixel super resolution to ***only one*** of the measurement heights or angles, which is used as an initial guess at Stage I of our reconstruction algorithm detailed in the Methods Section. As will be presented next, this approach is also quite efficient in terms its data requirement compared to existing approaches.

### Improving the Data Efficiency in High-resolution Holographic Reconstructions Using the Propagation Phasor Approach

Using the multi-height imaging configuration outlined earlier, we performed 4 × 4 lateral shift-based pixel super-resolution at *only one* sample-to-sensor distance (i.e., ~190 μm), which added 15 extra raw measurements/holograms to the original data set that is composed of measurements at 8 heights. In our propagation phasor based reconstruction, we directly used the back-propagation of this super-resolved hologram at this height (190 μm) as our initial guess (Stage I of our algorithm – see the Methods Section). The resolution improvement that we have got by using these additional 15 raw measurements in our propagation phasor approach is significant: we achieved a half-pitch resolution of ~0.55 μm (corresponding to an NA of ~0.48 at 530 nm illumination), which is the same level of resolution that is achieved by performing lateral shift-based super-resolution *at every height* (see Fig. 6a,b). In other words, to achieve the same resolution level, the propagation phasor approach utilized ***5.5-fold*** less number of raw measurements (i.e., 23 vs. 128) compared to the conventional lateral shift-based multi-height method^25^,^27^,^32^.

A similar level of improvement in data efficiency of our propagation phasor approach is also observed in the multi-angle imaging configuration: by simply performing 6 × 6 pixel super-resolution at *only* the vertical illumination, the propagation phasor based reconstruction can achieve a half-pitch resolution of ~0.49 μm (corresponding to an NA of ~0.53 at 530 nm illumination). As a comparison, the synthetic aperture approach^33^ achieves a half-pitch resolution of ~0.44 μm; however it uses 6 × 6 pixel super-resolution *at every* illumination angle (Fig. 6c), and therefore our propagation phasor approach (Fig. 6d) has ***7-fold*** improvement in its data efficiency (i.e., 44 vs. 324 raw measurements). This improvement and significant reduction in the number of raw measurements/holograms are especially important to make wide-field, high-resolution holographic imaging suitable for high speed applications.

### Imaging Biological Samples Using the Propagation Phasor Approach

**Figure 7:**
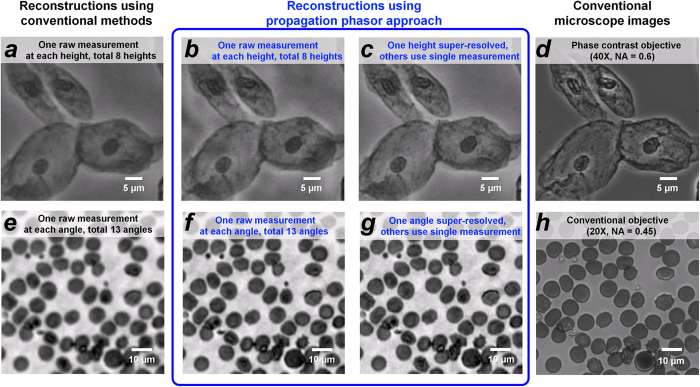
Holographic imaging of biological samples using the propagation phasor approach. First row: imaging of an unstained Pap smear; second row: imaging of a stained blood smear. (**a**) Conventional multi-height based reconstruction^25^. (**b**) Propagation phasor approach-based reconstruction using the same data set used in (**a**). (**c**) Propagation phasor approach-based reconstruction, where at one of the heights (150 μm), 9 raw measurements are used for pixel super-resolution, while at each of the other heights, only one raw measurement is used. (**d**) Conventional phase contrast microscope image of the same sample using a 40× objective lens (NA = 0.6). (**e**) Conventional synthetic aperture based reconstruction^33^. At each of the 13 illumination angles (−30° : 10° : 30°, two axes), only one raw measurement is used. (**f**) Propagation phasor approach-based reconstruction using the same data set used in (**e**). (**g**) Propagation phasor approach-based reconstruction, where at the normal illumination, 9 raw measurements are used for pixel super-resolution, while at each one of the other angles, only one raw measurement is used. (**h**) Conventional bright-field microscope image of the same blood smear using a 20× objective lens (NA = 0.45).

To demonstrate the success of our propagation phasor approach in imaging biological specimen, we imaged unstained Papanicolaou (Pap) smears (see Fig. 7a–d) and stained blood smears (see Fig. 7e–h). For Pap smear imaging, we captured the holograms of the specimen at multiple sample-to-sensor distances, and at each *z*_*k*_, only one raw measurement is recorded. For comparison, we first reconstructed the Pap smear using a previously reported multi-height phase retrieval algorithm^25^,^27^,^32^ (Fig. 7a). Using the same holographic data set and raw measurements, the reconstructions created by our propagation phasor approach (Fig. 7b) show resolution improvements compared to the previously reported method. To further improve the resolution without significantly increasing the burden of data acquisition, we added eight extra raw measurements for shift-based pixel super-resolution (with a super-resolution factor of 3 × 3) at *only one* of the heights, which is used as an initial guess (in Stage I) of our reconstruction algorithm. As shown in Fig. 7c, our propagation phasor approach based reconstruction shows a good agreement with the images captured using a conventional phase contrast microscope (40 × objective lens, NA = 0.6). For imaging of stained blood smears, we captured the lensfree holograms at multiple illumination angles. The comparison between Fig. 7e and Fig. 7f also confirms the resolution improvement brought by our propagation phasor based reconstruction algorithm. By adding lateral shift-based pixel super-resolution (with a super-resolution factor of 3 × 3) at only the vertical illumination angle (i.e., *θ*_*k*_ = 0), we further improved the resolution of our reconstructed image (Fig. 7g), which shows comparable performance against a bright-field microscope with a 40 × objective lens (NA = 0.6), Fig. 7h.

Based on these results, we confirm that our propagation phasor approach would greatly increase the speed of high-resolution and wide-field holographic microscopy tools. In previously reported holographic imaging modalities, multiple laterally shifted images are captured to achieve pixel super-resolution at every one of the sample-to-sensor distances^25^,^27^,^32^ or illumination angles^33^. As demonstrated in Figs 6 and 7, the propagation phasor approach can reduce the number of required raw holograms by five to seven fold while also achieving a competitive resolution. This reduction in raw data also lowers the need for data transmission and storage, which could further improve the cost-effectiveness of holographic imaging modalities such as handheld lensfree microscopy tools^22^,^27^,^34^ for telemedicine applications.

Although our experimental demonstrations in this manuscript utilized a lensfree on-chip imaging set-up, we would like to once again emphasize that this propagation phasor approach is broadly applicable to a wide range of holographic imaging modalities, including lens-based holographic microscopy techniques. For instance, in a lens-based undersampled holographic imaging system, multi-height pixel super-resolution can simply be achieved by capturing a series of defocused images at different heights. Considering the fact that the depth focusing operation is naturally required and performed every time a sample is loaded onto a lens-based traditional microscope, this propagation phasor approach provides a unique method to enlarge the space-bandwidth-product of the final image without compromising the image acquisition time.

## Conclusions

In this manuscript, we demonstrated a propagation phasor approach for high-resolution, wide-field holographic imaging with significantly improved data efficiency. Different from previous holographic reconstruction methods, our propagation phasor approach merges phase retrieval and pixel super-resolution techniques into a unified mathematical framework, where the twin image noise, spatial aliasing signals and other digital artifacts are all interpreted as noise terms that are modulated by phasors. These propagation phasors analytically depend on and can be controlled by various imaging parameters such as the lateral displacement between the hologram and the sensor-array, sample-to-sensor distance, illumination wavelength, and the angle of incidence. We systematically investigated and summarized the sensitivities of both the twin image noise and the aliasing signal to these imaging parameters, which enabled us to establish two new super-resolution methods that utilize the diversity of the sample-to-sensor distance and the diversity of the illumination angle. Compared to previous reconstruction algorithms, this propagation phasor framework can deliver phase-retrieved reconstructions with a competitive resolution using five- to seven-fold reduced number of raw measurements/holograms, which makes it especially appealing for high speed and cost effective microscopy applications. We further confirmed the success of this approach by imaging biological samples including unstained Papanicolaou smears and stained blood smears.

## Additional Information

**How to cite this article**: Luo, W. *et al.* Propagation phasor approach for holographic image reconstruction. *Sci. Rep.* **6**, 22738; doi: 10.1038/srep22738 (2016).

## Supplementary Material

Supplementary Information

## Figures and Tables

**Table 1:** Summary of the methods that can be used for the elimination of twin image noise and spatial aliasing signal terms in digital holographic imaging.

	Lateral shifts *x*_*shift,k*,_ *y*_*shift,k*_	Sample-to-sensor distance *z*_*k*_	Illumination wavelength *λ*_*k*_	Illumination angle *θ*_*k*_*, φ*_*k*_
Twin image noise elimination	Not applicable	*Method*: Multi-height based phase retrieval *(e.g., Refs* 13,25,27,32,36,37,49,50)	*Method*: Multi-wavelength based phase retrieval *(e.g., Refs* 46,55)	*Method*: Synthetic aperture *(e.g., Ref.* 33)
Aliasing signal elimination	*Method*: Lateral shift-based pixel super-resolution *(e.g., Refs* 16,22,25,26,30, 31, 32, 33,40, 41, 42, 43,54)	Multi-height based pixel super-resolution *(This paper)*	*Method*: Wavelength-scanning pixel super-resolution *(e.g., Ref.* 35)	Multi-angle based pixel super-resolution *(This paper)*
