# Corrigendum: Propagation phasor approach for holographic image reconstruction

**DOI:** 10.1038/srep34679

**Published:** 2016-10-19

**Authors:** Wei Luo, Yibo Zhang, Zoltán Göröcs, Alborz Feizi, Aydogan Ozcan

Scientific Reports 6: Article number: 2273810.1038/srep22738; published online: 03 11 2016; updated: 10 19 2016

Here we correct and clarify some of the mathematical expressions in this Article. These do not affect the conclusions and the reported results of the original published article. In the Methods section under the subheading ‘Mathematical formalism of propagation phasor approach in digital holography’,

“*SS*_*k*_ represents the self-interference terms, which can be written as 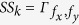
{*H*_*k*_  ·  *S*_*k*_}, where 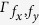 refers to the autocorrelation operation”.

should read:

*“SS*_*k*_ represents the self-interference terms, which can be written as 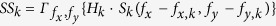, where 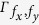 refers to the autocorrelation operation”.

In the same section Equation 4,


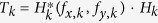


should read:


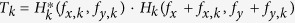


In the same section under the subheading ‘Stage I of Propagation Phasor based Holographic Reconstruction: Generation of an Initial Guess’,

“The right side of Eq. (9) shows that in order to extract (*δ*_00_ + *S*_00,*k*_ · *P*_00,*k*_, there are five terms that need to be eliminated from the back-propagated intensity (i.e., 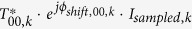)”.

should read:

“The right side of Eq. (9) shows that in order to extract (*δ*_00_ + *S*_00,*k*_) · *P*_00,*k*_, there are five terms that need to be eliminated from the back-propagated intensity (i.e., 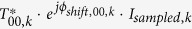)”.

