# Supplementary material for: Propagation phasor approach for holographic image reconstruction
Source: Sci Rep. 2016 Mar 11;6:22738. doi: 10.1038/srep22738 (PMC4786813; doi:10.1038/srep22738)
Supplement: Supplementary Information [file srep22738-s1.pdf]

Supplementary information for

Propagation phasor approach for holographic image reconstruction

Authors: Wei Luo[1,2,3], Yibo Zhang[1,2,3], Zoltan Göröcs[1,2,3], Alborz Feizi[1,2,3] and Aydogan Ozcan[1,2,3,4]

Affiliations:

[1]Electrical Engineering Department, University of California, Los Angeles, CA, 90095, USA.

[2]Bioengineering Department, University of California, Los Angeles, CA, 90095, USA.

[3]California NanoSystems Institute (CNSI), University of California, Los Angeles, CA, 90095, USA.

[4]Department of Surgery, David Geffen School of Medicine, University of California, Los Angeles, CA, 90095, USA.

Correspondence: Prof. Aydogan Ozcan
E-mail: ozcan@ucla.edu
420 Westwood Plaza, Engr. IV 68-119, UCLA
Los Angeles, CA 90095, USA
Tel: +1(310)825-0915, Fax: +1(310)206-4685

Authors' email addresses:

Wei Luo: luow@ucla.edu
Yibo Zhang: zybmax@g.ucla.edu
Zoltan Göröcs: zoli.gorocs@gmail.com
Alborz Feizi: alborz.fe@ucla.edu
Aydogan Ozcan: ozcan@ucla.edu

Mathematical relationship between the propagation phasor approach-based initial guess and the back-propagation of a pixel super-resolved hologram

From (Eq. 13) of the main text, the initial guess for the specimen (Stage I of our propagation phasor approach) can be written as:

$$(\delta_{00} + S_{00,\,initial}) \cdot P_{00} \equiv \frac{1}{K}\sum_{k} T^{*}_{00,\,k} \cdot e^{j\phi_{shift,\,00,\,k}} \cdot I_{sampled,\,k} \qquad \text{S1}$$

Recall the expression of $I_{sampled,\,k}$ from (Eq. 6) of the main text:

$$I_{sampled,\,k} = \sum_{u,v=0,\pm1,\pm2,\cdots} I_{uv,\,k} \cdot P_{uv,\,k} \cdot e^{-j\phi_{shift,\,uv,\,k}} \qquad \text{S2}$$

By replacing $I_{sampled,\,k}$ in Eq. S1 with the expression of S2, we can get:

$$\begin{aligned} &\frac{1}{K}\sum_{k} T^{*}_{00,\,k} \cdot e^{j\phi_{shift,\,00,\,k}} \cdot I_{sampled,\,k} \\ &= \frac{1}{K}\sum_{k}\sum_{u,v} T^{*}_{00,\,k} \cdot e^{j\phi_{shift,\,00,\,k}} \cdot I_{uv,\,k} \cdot P_{uv,\,k} \cdot e^{-j\phi_{shift,\,uv,\,k}} \end{aligned} \qquad \text{S3}$$

Notice that the diversity of the lateral shifts does not affect the transmission properties of the sample, the pixel function $P_{uv,\,k}$, or the propagation phasor $T^{*}_{00,\,k}$, and therefore we have $I_{uv,\,k} = I_{uv}$, $P_{uv,\,k} = P_{uv}$, and $T^{*}_{00,\,k} = T^{*}_{00}$ for all K measurements. Based on this, S3 can be expressed as:

$$\frac{1}{K}\sum_{k} T^{*}_{00,\,k} \cdot e^{j\phi_{shift,\,00,\,k}} \cdot I_{sampled,\,k} = \frac{1}{K} T^{*}_{00} \sum_{u,v} I_{uv} \cdot P_{uv} \sum_{k} e^{j\left(\phi_{shift,\,00,\,k} - \phi_{shift,\,uv,\,k}\right)} \qquad \text{S4}$$

If the lateral shifts are evenly distributed within one pixel pitch, where $x_{shift,\,k} \in \{m/(M\cdot\Delta x) \mid m = 1,2,...,M\}$ and $y_{shift,\,k} \in \{n/(N\cdot\Delta y) \mid n = 1,2,...,N\}$, then we have in total $M\times N=K$ measurements. For convenience of our derivations, we will replace the index k with (m, n), and rewrite $\phi_{shift,\,uv,\,k}$ as $\phi_{shift,\,uv,\,mn}$ i.e.,

$$\phi_{shift,\,uv,\,mn} = 2\pi[(f_x - \frac{u}{\Delta x}) \cdot m \cdot \frac{\Delta x}{M} + (f_y - \frac{u}{\Delta x}) \cdot n \cdot \frac{\Delta y}{N}] \qquad \text{S5}$$

Therefore, we have:

$$\phi_{shift,\,00,\,mn} - \phi_{shift,\,uv,\,mn} = 2\pi[\frac{u \cdot m}{M} + \frac{v \cdot n}{N}] \qquad \text{S6}$$

The summation $\sum_k e^{j\left(\phi_{shift,\,00,\,k} - \phi_{shift,\,uv,\,k}\right)}$ in Eq. S4 can be rewritten as $\sum_{m,n} e^{j\left(\phi_{shift,\,00,\,mn} - \phi_{shift,\,uv,\,mn}\right)}$, and

$$\begin{aligned} &\sum_{m,n} e^{j\left(\phi_{shift,\,00,\,mn} - \phi_{shift,\,uv,\,mn}\right)} = \sum_{m,n} e^{j2\pi[\frac{u \cdot m}{M} + \frac{v \cdot n}{N}]} \\ &= \sum_{m=1}^{M} e^{j2\pi\frac{u \cdot m}{M}} \cdot \sum_{n=1}^{N} e^{j2\pi\frac{v \cdot n}{N}} = \begin{cases} M \cdot N & u = 0 \text{ and } v = 0 \\ 0 & \text{else} \end{cases} \end{aligned} \qquad \text{S7}$$

Therefore, the right side of Eq. S4 becomes

$$\begin{aligned} &\frac{1}{K} T_{00}^* \sum_{u,v} I_{uv} \cdot P_{uv} \sum_k e^{j\left(\phi_{shift,\,00,\,k} - \phi_{shift,\,uv,\,k}\right)} = \frac{1}{K} T_{00}^* \cdot I_{00} \cdot P_{00} \cdot M \cdot N \\ &= T_{00}^* \cdot I_{00} \cdot P_{00} \end{aligned} \qquad \text{S8}$$

Eventually, we arrive at:

$$\begin{aligned} &(\delta_{00} + S_{00,\,initial}) \cdot P_{00} \equiv \frac{1}{K} \sum_k T_{00,\,k}^* \cdot e^{j\phi_{shift,\,00,\,k}} \cdot I_{sampled,\,k} \\ &= T_{00}^* \cdot I_{00} \cdot P_{00} \end{aligned} \qquad \text{S9}$$

where T_{00}^* is the back-propagation phasor, and I_{00} is the high-resolution hologram. ***Therefore, we can conclude that when the raw measurements are laterally shifted holograms, where the sub-pixel shifts are uniformly distributed within a pixel, the initial guess generated by using our propagation phasor approach (Stage I) is mathematically equivalent to the back-propagation of a pixel super-resolved hologram.***
